# Supplementary material for: Freezing and thawing magnetic droplet solitons
Source: Nat Commun. 2022 May 5;13:2462. doi: 10.1038/s41467-022-30055-7 (PMC9072373; doi:10.1038/s41467-022-30055-7)
Supplement: Supplementary file 1 — Supplementary information [file 41467_2022_30055_MOESM1_ESM.pdf]

Supplementary information for
Freezing and thawing magnetic droplet solitons

Martina Ahlberg[1,*], Sunjae Chung[1,2,*,+], Sheng Jiang[1,3,4], Andreas Frisk[1], Maha Khademi[5], Roman Khymyn[1], Ahmad A. Awad[1], Q. Tuan Le[1,4], Hamid Mazraati[4,6], Majid Mohseni[4,5], Markus Weigand[7], Iuliia Bykova[7], Felix Groß[7], Eberhard Goering[7], Gisela Schütz[7], Joachim Gräfe[7], & Johan Åkerman[1,4,+]

[1]*Department of Physics, University of Gothenburg, 412 96 Gothenburg, Sweden*

[2]*Department of Physics Education, Korea National University of Education, Cheongju 28173, Korea*

[3]*School of Microelectronics, Northwestern Polytechnical University, Xi'an 710072, China*

[4]*Department of Applied Physics, School of Engineering Sciences, KTH Royal Institute of Technology, 100 44 Stockholm, Sweden*

[5]*Department of Physics, Shahid Beheshti University, Evin, 1983969411 Tehran, Iran*

[6]*NanOsc AB, 164 40 Kista, Sweden*

[7]*Max Planck Institute for Intelligent Systems, Stuttgart, Germany*

[*]*These authors contributed equally to this work.*

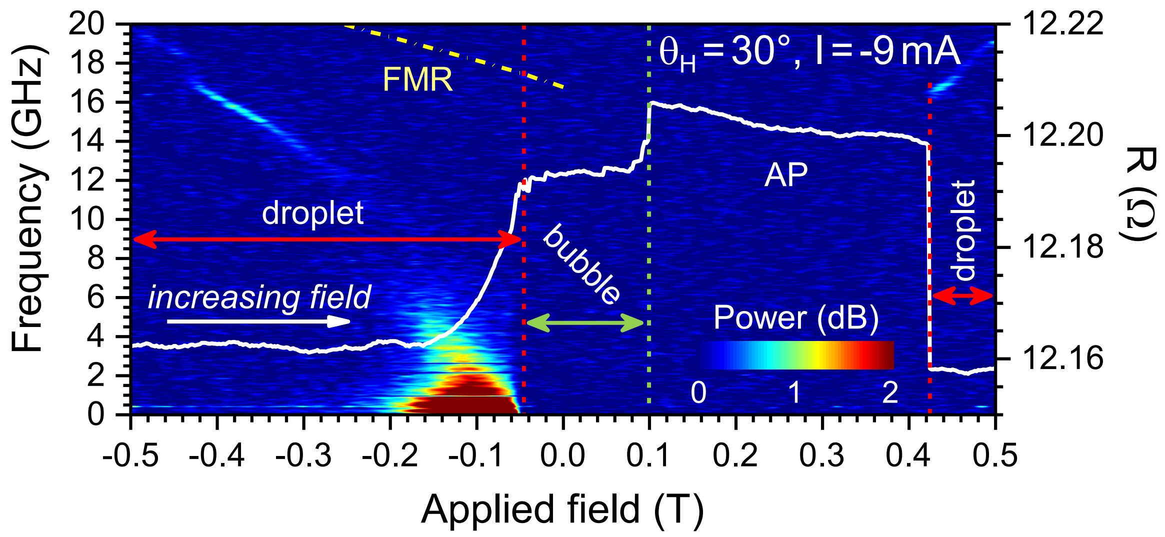

Figure S1: **Supplementary Fig. 1.** Color plot of the power spectral density (PSD) of the microwave noise as a function of increasing field, with the STNO resistance (white line) overlayed; the applied current is -9 mA and the field angle is $\Theta_{\mathrm{H}} = 30°$ from the film plane. AP indicates the antiparallel state of the STNO; red arrow indicates the droplet region, and green arrow the bubble region. The yellow dashed-dotted line shows the calculated FMR using the gyromagnetic ratio $\gamma/2\pi = 30.5$ GHz/T, uniaxial anisotropy $K_{\mathrm{u}} = 519$ kJ/m^3 and saturation magnetization $M_{\mathrm{s}} = 716.2$ A/m.

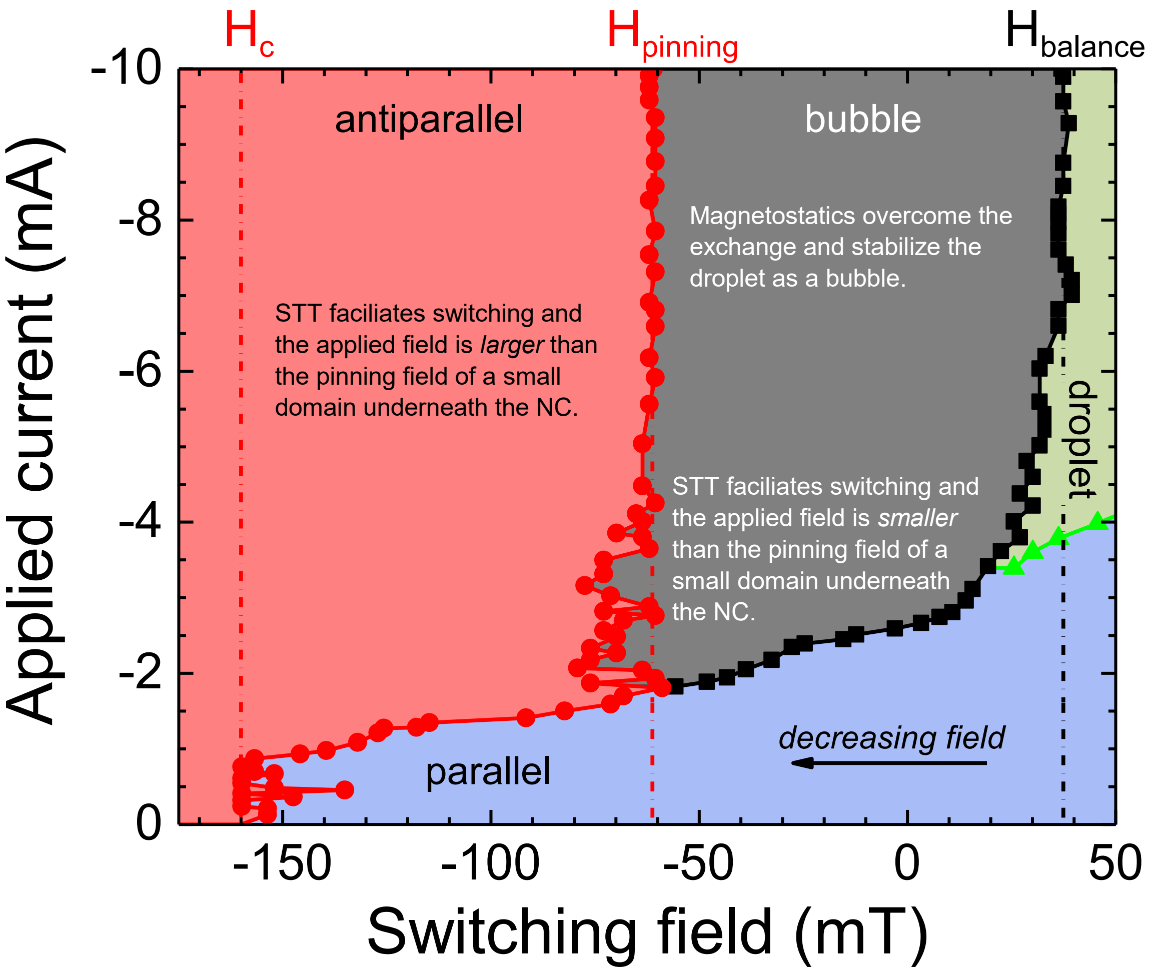

Figure S2: **Supplementary Fig. 2.** A zoom-in of the phase diagram in Fig. 3(a) of the main text. The color code represents the different states: droplet (green), bubble (gray) and antiparallel (red). A droplet is nucleated at high currents and fields. Below a certain positive field (H_{balance}) the droplet is stabilized as a static bubble due to magnetostatic effects[1]. The bubble is pinned below the nanocontact until the negative field is high enough to let the bubble domain expand throughout the film at H_{pinning}. At low currents the magnetic switching is only governed by the coercive field (H_{c}) of the free layer.

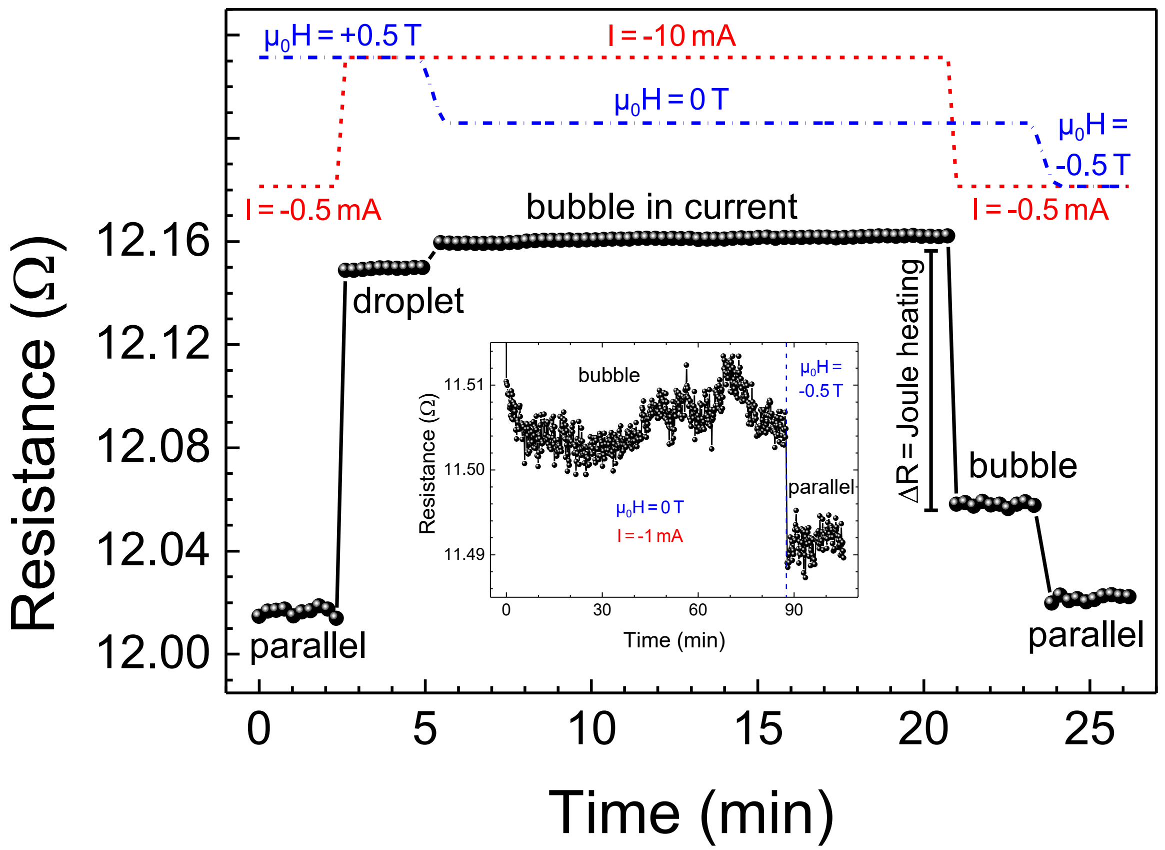

Figure S3: **Supplementary Fig. 3.** Resistance as a function of time. The top red dashed and blue dash-dotted lines illustrate how the current and field, respectively, are varied during the experiment. The initial high field ($+0.5$ T) and very low current establish a simple parallel state. After 2.5 minutes the current is increased to -10 mA and a droplet is nucleated. Thereafter the field is set to zero and a bubble is formed. The bubble remains intact after the current is practically switched off ($I = -0.5$ mA). The large difference in resistance is caused by the strong reduction of the Joule heating. A strong opposite field erases the bubble and resets a parallel state. The inset presents a longer measurement of the bubble stability in zero field and virtually zero current.

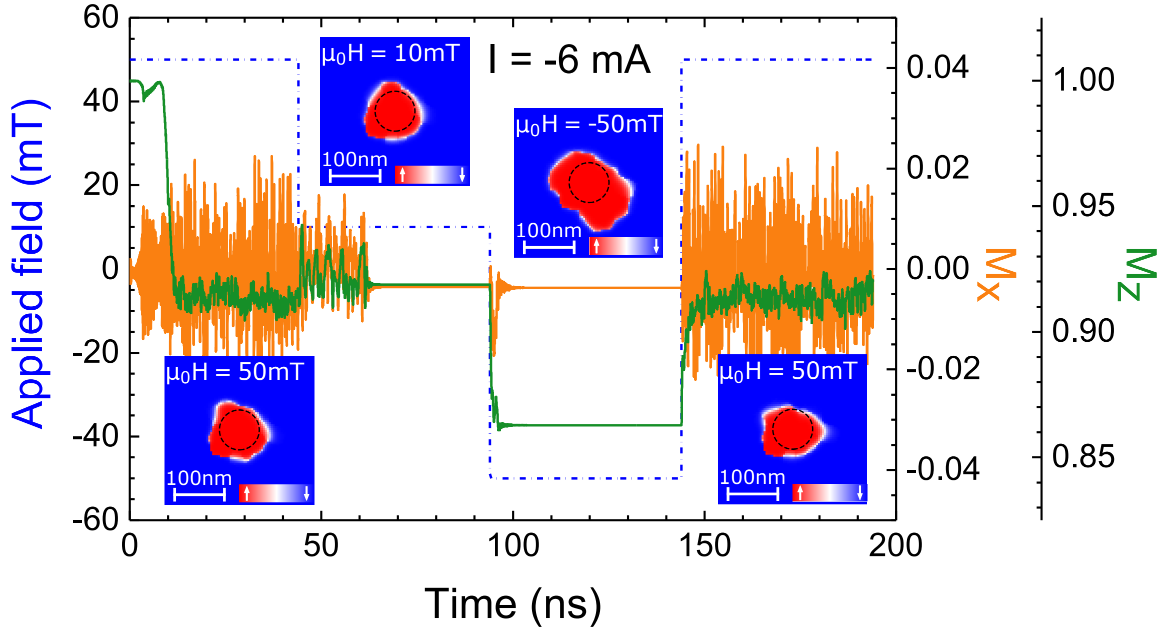

Figure S4: **Supplementary Fig. 4.** Results of micromagnetic simulations. The applied current is constant, $I = -6$ mA, while the field is varied in steps presented by the blue dashed-dotted line. The orange and green solid lines represent the x- and z-component of the magnetization, respectively, and illustrate the spin dynamics as a function of time and field. The full magnetization vector, $\mathbf{M} = (M_\mathrm{x}, M_\mathrm{y}, M_\mathrm{z})$, consists of the mean values over the whole simulated volume, normalized to one. At a moderate field of 50 mT, a wobbly but well-defined droplet is nucleated. When the field is reduced to 10 mT, the droplet freezes into a bubble within 20 ns. The bubble grows when the field is further reduced to -50 mT. The dynamic droplet mode is instantly restored when the field is reset to 50 mT. The insets show snapshots of the magnetic state at each field.

1. Hoefer, M. A., Silva, T. J. & Keller, M. W. Theory for a dissipative droplet soliton excited by a spin torque nanocontact. *Phys. Rev. B* **82**, 054432 (2010).
